# Commentary: Elimination of Left-Right Reciprocal Coupling in the Adult Lamprey Spinal Cord Abolishes the Generation of Locomotor Activity

**DOI:** 10.3389/fncir.2018.00034

**Published:** 2018-04-25

**Authors:** Lorenzo Cangiano, Sten Grillner

**Affiliations:** ^1^Department of Translational Research, University of Pisa, Pisa, Italy; ^2^Department of Neuroscience, Karolinska Institute, Stockholm, Sweden

**Keywords:** locomotion, central pattern generators, oscillators, coordination, coupling, lamprey, spinal cord, rhythmicity

We like to comment on the article by Messina et al. (2017) entitled “*Elimination of left-right reciprocal coupling in the adult lamprey spinal cord abolishes the generation of locomotor activity*.” That a removal of the connection between the left and right side eliminates coordinated locomotion is obvious. But their additional claim is that each side of the spinal cord is unable to generate the rhythmic locomotor-related activity. We, on the other hand, in previous experiments have shown that swimming is “*produced by pairs of unilateral burst generating networks with reciprocal inhibitory connections that not only ensure left/right alternation but also downregulate frequency”* (Cangiano and Grillner, 2003, 2005; Cangiano et al., 2012). We find that the results of Messina et al. are well compatible with our findings, however, very surprisingly they reach another conclusion.

In previous reports (Cangiano and Grillner, 2003; Cangiano, 2004) we show that during ongoing fictive locomotion, recorded in the ventral roots, a progressive series of small well-controlled midline lesions will lead to a progressive increase of the burst frequency until the two halves become entirely separated (Figure 1A). It is important to note that this links the burst-activity in the two separate spinal cord halves to that of the locomotor-activity in the intact cord, and that in all transitional phases there are distinct periodic bursts. Similarly, a bout of locomotor burst-activity can be initiated by stimulation of the rostral end of the hemicord and well-coordinated bursting will be set up along the hemicord (Cangiano and Grillner, 2005). Particularly toward the last part of the locomotor bout (Figure 1B), the frequency overlaps with the range seen in the intact swimming lamprey. The capacity of the hemicord to generate locomotor-related activity is manifested directly after the midline section is completed (Cangiano et al., 2012).

**Figure 1:**
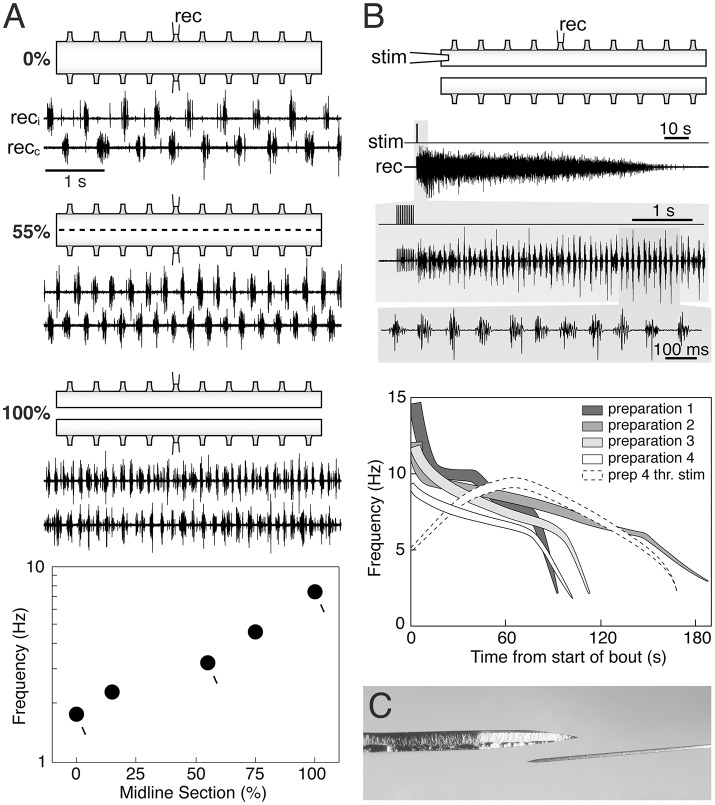
The hemi-spinal cord of the lamprey is capable of expressing locomotor-related rhythmic bursting. **(A)** When activated pharmacologically (0.75 mM D-glutamate in this panel) intact *ex vivo* spinal cord preparations express the left-right alternating rhythmic pattern of fictive swimming (0%). Reducing crossed connections by 55% with intermittent midsagittal sections increases burst frequency while maintaining alternation. Complete separation of the two hemicords (100%) leads to relatively fast autonomous bursting on each side. A plot of burst frequency vs. extent of separation shows that fictive swimming in the intact cord and rhythmic bursting in the hemicord are functionally linked, with the latter representing the operation of the locomotor network in the absence of crossed connections. **(B)** The hemicord is able to express longlasting bouts of rhythmic bursting simply in response to brief electrical stimulation (i.e., without glutamatergic agonists) (upper traces). Burst frequencies vary in a wide range that partially overlaps with that of swimming (silhouettes in the graph). Higher frequencies correlate with stronger levels of network activity (thickness of the silhouettes). Weak threshold electrical stimulation evokes self-reinforcing activity (dashed white silhouette), demonstrating the intrinsic excitability of the hemicord when deprived of contralateral inhibition. Adapted with permission from Cangiano and Grillner (2003, 2005). **(C)** Dorsal-view of the Beaver mini-blade 376500 used by Messina et al. (2017) to hemisect the spinal cord (above), next to an ophthalmic knife of the type used in our experiments (below). A thicker blade is likely to cause more lateral compression in the tissue being cut.

Messina and colleagues recorded the EMG activity evoked by stimulation of the oral hood in *in vivo* preparations before and after performing a midline section of the rostral spinal cord, followed by a caudal transection, leaving the two hemicords connected to the brainstem, but otherwise separated. They reported that EMG activity was frequently tonic/unpatterned when the oral hood was stimulated, but in all preparations 30% of the stimulation trials evoked bursting at frequencies from the upper range of swimming to higher values. Under their experimental conditions the hemicords were able to express a relatively fast rhythmic motor output, similar to our *ex vivo* preparations directly linked to fictive swimming in the intact cord (Figure 1A). Messina et al. did not, in contrast to Cangiano and Grillner (2003), establish this link experimentally nor did they interpret their results on the basis of our aforementioned work.

A likely explanation for the somewhat higher maximum frequencies reported by Messina et al. relative to those expressed in our *ex vivo* hemicords, is that in their casedescending input from the brainstem acted as a supplementary drive to the already highly excitable hemicords (Cangiano and Grillner, 2005; Huss et al., 2006). As for the burst proportion, the observation of a higher value compared to that during swimming is not surprising given that in the intact cord the left and right sides do receive phasic contralateral inhibition. Reciprocal inhibition not only coordinates the two sides, but also lowers the frequency of bursting and shortens the bursts (burst proportion) on each side (Cangiano and Grillner, 2005).

In our hands rhythmicity is expressed reliably and with a good-excellent quality (Figure 1), provided that the sectioning along the midline is performed with great caution under the microscope, using thin ophthalmic knifes mounted on a manipulator to make successive accurate lesions (references above). Messina et al. do not provide details with regard to the process of midline sectioning except that it was performed *in vivo* with a Beaver mini-blade (Figure 1C) and that the wound was closed afterwards and the animal returned to a container. It should be noted that tonic activity had also been observed in isolated hemicords before our original report (Cangiano and Grillner, 2003) that we then ascribed to a less precise midline sectioning (Cangiano, 2004). Furthermore, we are unconvinced by the argument that the presence of left-right bursting in midline-only preparations (intact caudal spinal cord) should be indicative of the absence of a scalpel or glue induced injury, since the activity must have been exogenously driven from the intact part of the spinal cord (as shown by its disappearance after transection).

One further difference is that our studies were performed on isolated spinal hemicords, while that of Messina et al. was done in behaving lampreys subjected to a midline incision with EMG activity/swimming initiated by oral hood stimulation. Moreover, all sensory feedback and segmental activity was presumably in operation. The sensory feedback from erratic contractions on the two sides could well affect the ventral root activity. We find the isolated condition being a much cleaner experimental situation to investigate the ability of the hemi-spinal cord to generate unilateral bursting.

In conclusion, from the perspective of understanding the locomotor CPG, a critical finding is that after a midline lesion the unilateral spinal cord circuitry can generate rhythmic bursting, as shown by Cangiano and Grillner (2003, 2005) and Cangiano et al. (2012) and also by Messina et al. Normally, however, the burst generating networks on each side of the cord are coordinated through reciprocal inhibition. When the reciprocal inhibition has been blocked, the intact spinal cord will instead generate simultaneous bursting on both sides, resulting from the commissural excitatory neurons (Cohen and Harris-Warrick, 1984). This finding further emphasizes the fundamental fact that the ability to generate burst activity does not depend on reciprocal inhibition. From this follows, that the claim of Messina et al. that the burst-generation is crucially dependent on reciprocal inhibition is incorrect. Their data, as well as ours support independent burst generating networks, although of course dependent on reciprocal inhibition for the generation of actual locomotion. Moreover, postinhibitory rebound due to crossed inhibition can under some conditions promote bursting (Cangiano and Grillner, 2005; Wang et al., 2011; Moult et al., 2013).

## Author contributions

All authors listed have made a substantial, direct and intellectual contribution to the work, and approved it for publication.

### Conflict of interest statement

The authors declare that the research was conducted in the absence of any commercial or financial relationships that could be construed as a potential conflict of interest. The reviewer HH and handling Editor declared their shared affiliation.
